# Effect of Organic Compounds and Alkalinity on the Stability of Bulk Nanobubbles: A Molecular Dynamics Study

**DOI:** 10.3390/molecules30244712

**Published:** 2025-12-09

**Authors:** Samal Kaumbekova, Serina Ng, Dhawal Shah, Ayaulym Amankeldiyeva, Sagyn Omirbekov, Yanwei Wang

**Affiliations:** 1 Center for Energy and Advanced Materials Science, National Laboratory Astana, Nazarbayev University, 53 Kabanbay Batyr Avenue, Astana 010000, Kazakhstan; skaumbekova@alumni.nu.edu.kz (S.K.);; 2 Shijiazhuang Chang’An Yucai Building Materials Co., Ltd., Shijiazhuang 051430, China; serina.ng@cayc.info; 3 Department of Chemical and Materials Engineering, School of Engineering and Digital Sciences, Nazarbayev University, 53 Kabanbay Batyr Avenue, Astana 010000, Kazakhstan; dhawal.shah@nu.edu.kz

**Keywords:** bulk nanobubbles, nanobubble stability, intermolecular interactions, hydrophobic effects, solvent effects, gas solubility, molecular dynamics simulations

## Abstract

Bulk nanobubbles (NBs) are remarkably long-lived in liquids, yet the molecular mechanisms underpinning their stability remain unresolved. In this work, 50 ns all-atom molecular dynamics simulations were performed to investigate how gas identity (O_2_, N_2_, and air with N_2_:O_2_ = 4:1), initial gas loading, alkalinity (pH 7 and 13), and organic additives (acetic acid/acetate, ethanol/ethoxide, and hexane) influence the stability of 5 nm NBs in water. Stability was evaluated by the percentage of gas atoms retained in the bubble, density profiles, hydrogen-bond statistics, and radial distribution functions. Higher initial gas density markedly enhanced stability, and N_2_-NBs consistently outperformed O_2_-NBs, consistent with the lower solubility of N_2_. Alkaline conditions exerted only a minor stabilizing effect, most pronounced for air-NBs. Organic additives affected stability according to their hydrophobicity: hydrophobic hexane substantially increased gas retention, especially at low gas loading, by promoting gas clustering and re-adsorption at the NB interface, whereas hydrophilic solutes had negligible influence. RDF analyses revealed that this stabilization correlates with weakened gas–water hydrogen bonding and enhanced gas–gas and gas–hexane interactions. These results elucidate the molecular determinants of NB persistence and offer design guidelines for tuning bubble longevity in environmental and industrial systems.

## 1. Introduction

According to their size, the bubbles are usually divided into several groups, such as macrobubbles (diameter of 100–10,000 µm), microbubbles (10–100 µm), sub-microbubbles (1–10 µm), and nanobubbles (NBs, ultrafine bubbles, 0.001–1 µm or 1–1000 nm) [1]. Among the bubbles of different sizes, NBs are of high interest. The main unique property of NBs is their stability. In particular, NBs can remain stable in liquid for several weeks or months, whereas ordinary bubbles of a larger size would quickly disappear by rising to the surface of the liquid and collapsing with each other [2]. Moreover, NBs might be classified as bulk NBs, surface NBs, and micropancakes, depending on their location and the way they are generated [3,4].

NBs have attracted significant interest in various fields, including medicine, environmental science, and industry. For example, NBs might be used in wastewater treatment, improving aeration systems, biological oxygen demand (BOD), and chemical oxygen demand (COD) [5]. Furthermore, NBs might be used in agriculture and horticulture by improving the irrigation water quality, allowing crops to absorb more nutrients, and reducing the risk of diseases that lead to crop loss while reducing chemical applications [6]. NBs might be used for enhanced surfactant remediation of diesel-contaminated soil [7], enhanced oil recovery [8], oil–water separation [9], protein folding [10], and surface cleaning [11]. Micro-nanobubbles can also be used to improve the properties of concrete, such as tensile strength, compressive strength, concrete resistivity, and water absorption [12].

The NB stability depends on various factors, such as pH [13], ionic composition [14,15], and temperature [16], indicating the importance of optimal conditions for the effective usage of NBs. The experimental studies suggested that an alkaline environment with low pH might enhance the stability of NBs [13]. Similarly, according to Ke et al. [17], high stability of nitrogen NBs was observed in a basic environment (pH = 9.5), in comparison to an acidic environment (pH = 4.5), water, and NaCl (pH = 7). According to Ulatowski et al. [18], hydroxide ions on the NB surface have the effect of lowering the surface tension. The experimental study of Jin et al. [14] further revealed the formation of stable NBs in water in the presence of water-soluble organic molecules (α-cyclodextrin), with reduced surface tension of NBs.

Among different multiscale simulations, Molecular Dynamics (MD) simulations can be used to systematically investigate the properties and stability of NBs under different conditions. For example, according to the results of the MD simulations of Hewage et al. [16], higher initial gas density in NBs and system gas supersaturation are critical for stable NB conditions. Wang et al. [19] showed that the formation and stability of NBs might be controlled by regulating the sizes and periods of confinement of the hydrophobic nanopatterns. In particular, nitrogen NBs (N_2_-NBs) in water preferentially sit on hydrophobic domains rather than on hydrophilic domains [19]. A similar observation was reported by the MD study of Zhang et al. [20], who found that the hydrophobic wall is favorable for the adsorption of gaseous molecules, which makes it easier to form surface NBs. Likewise, according to Jin et al. [21], the water film on the molybdenite surface was ruptured when N_2_-NB replaced water molecules by attaching to the surface. In contrast, the hydrophilic quartz surface remained stable, as the NB did not attach within the time frame of the MD simulations.

Considering the importance of the stability of NBs for their applications, this research focuses on the factors affecting the stability of NBs, considering the pH conditions, NB gas density variations, and the presence of organic compounds with different functional groups and hydrophobicity. In particular, this molecular modeling is aimed at investigating the stability of NB models at alkaline conditions (pH = 13), mimicking the pH conditions of concrete or groundwater environments. Moreover, the effects of the NB gas type and gas density were studied, varying the gas composition (O_2_, N_2_, air model) and gas concentration in the NBs. Furthermore, the stabilizing effects of co-solvents on NBs were examined using organic compounds with various functional groups. Specifically, acetic acid, ethanol, and hexane molecules were chosen, which might be further implicated in the applications of NBs in concrete admixture technology.

## 2. Methodology

*Gromacs 2022* software was used to perform MD simulations with the *OPLS-AA* forcefield parameters. The coordinates and topology of ions and organic compounds were taken from the ATB server [22]. The optimized forcefield parameters of O_2_ (δ = 0.34002 nm, ε = 0.49970) and N_2_ (δ = 0.40678 nm, ε = 0.59687 kJ/mol) were taken from the literature [23]. The simulations were performed in 10 × 10 × 10 nm^3^ boxes. The diameter of the initial bulk NB at the beginning of the simulations was 5 nm. The air-NB was modeled by keeping the molar ratio of N_2_ to O_2_ as 4:1 in the NB, taking into account only the two most abundant gases in the air model. The NB was solvated in water with an *SPC* water model. 60 OH^−^ ions were added to adjust alkaline conditions (pH 13). The simulation boxes were neutralized via Na^+^ ions.

### 2.1. Stability of Nanobubbles: Effect of NB Gas Type, Initial Gas Densities, and Alkalinity

**Figure 1:**
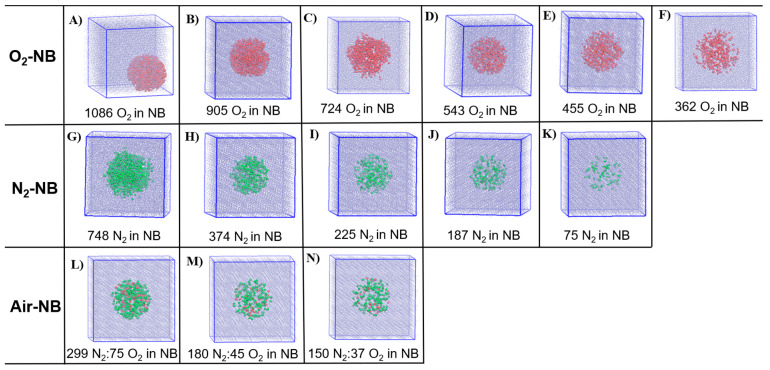
Representative snapshots of the simulation boxes at the beginning of the simulations: (**A**–**F**) O_2_-NB with different gas densities, (**G**–**K**) N_2_-NB with different gas densities, (**L**–**N**) Air-NB with different gas densities. Coloring methods: (1) Van der Waals representations: red—oxygen, green—nitrogen; (2) lines: blue—water molecules.

The number of molecules in the simulated systems is shown in Table 1. Keeping the initial NB diameter of 5 nm, the concentration of O_2_ in the NBs varied in the range between 38.4 g/L and 57.6 g/L, consistent with the estimated concentrations of O_2_ gas in NBs revealed from an experimental study [24]. Considering the lower solubility of N_2_ in water, the concentrations of N_2_ in the NBs varied in the range between 3.5 g/L and 34.8 g/L. Similarly, the concentrations of gas molecules in air-NBs varied in the range between 8.9 g/L and 17.9 g/L. The required number of molecules used in the simulated systems is shown in Table 1. The effect of alkaline conditions was studied at pH 13, inserting 60 OH^−^ ions and 60 Na^+^ ions (for a total neutral charge) into the simulation box. The visualized simulated systems with NBs of different gas concentrations at the beginning of the simulations are shown in Figure 1.

### 2.2. Effect of Organic Compounds with Various Functional Groups on the Stability of NB

The effect of organic compounds with various functional groups and hydrophobicity, such as carboxylic acid, alcohol, and alkane, on NB stability was investigated. In particular, the effect of acetic acid (with an n-octanol–water partition coefficient, log K_OW_ = −0.17, hydrophilic), ethanol (log K_OW_ = −0.30, hydrophilic), and hexane (log K_OW_ = 3.90, hydrophobic) molecules [25] on NB stability was studied. 100 organic molecules were randomly inserted into the simulation boxes with one bulk NB (d = 5 nm), as shown in Table 2 and visualized in Figure S1. It should be mentioned that the deprotonated forms of acetic acid and ethanol molecules were used at pH 13. Na^+^ ions were added to neutralize the total charge.

The effect of organic compounds was studied on O_2_-NBs and N_2_-NBs with high and low gas densities. In addition, the synergistic effect of organic compounds and pH condition variation (pH = 7, pH = 13) was further explored for O_2_-NB with 362 molecules in the initial NBs. Moreover, the effect of hexane molecules was studied on the air-NBs with different gas densities in the initial NB.

### 2.3. MD Simulations and Methods of the Analysis

To optimize the energy, the maximum atomic force constraint was set to 100 kJ mol^−1^ nm^−1^. Next, the constant-volume, constant-temperature (NVT) ensemble was run for 0.010 ns with H-bond constraints at a constant temperature of 298 K, with a time step of 0.0002 ps. The constant-pressure, constant-temperature (NPT) equilibration step was carried out for 0.10 ns, with a time step of 0.002 ps, using all-bonds constraints at a constant pressure and temperature of 1 bar and 298 K. The C-rescale barostat and Nose–Hoover thermostat were used for temperature and pressure couplings, respectively. The periodic boundary conditions in xyz-directions were used. The short-range interactions were defined using a 1 nm distance. The MD runs were performed for 50 ns with an integration time step of 0.002 ps, using the Nose–Hoover thermostat and Parrinello–Rahman barostat. During equilibration, the C-rescale barostat was used for smooth and stable pressure coupling to ensure accurate NPT ensemble sampling, followed by the Parrinello–Rahman barostat in the MD run for rigorous pressure control and correct ensemble fluctuations. Long-range electrostatics were treated using the Particle-Mesh Ewald (PME) method with a 1.0 nm real-space cutoff.

The results of the simulations and NB stability were analyzed via Gromacs analysis tools. Cluster analysis was performed to study the time evolution of the number of gas atoms in the NBs within the simulation, starting from the NVT step. In particular, a 1 nm cut-off distance between the center of masses of the atoms was specified to be considered a cluster. In addition, H-bonding and radial distribution function (RDF) analyses were performed to study the interactions between gas molecules and solvation in water. The obtained results were averaged over the last 10 ns of the MD runs when the simulated systems were stabilized. Moreover, the density analysis of NBs was performed for the first 4 ps and the last 4 ps of the simulations to study the NB size. The simulated systems were visualized using Visualized Molecular Dynamics (VMD) Gromacs 2022 software [26].

## 3. Results and Discussion

### 3.1. Stability of Nanobubbles: Effect of NB Gas Type, Initial Gas Densities, and Alkalinity

**Figure 2:**
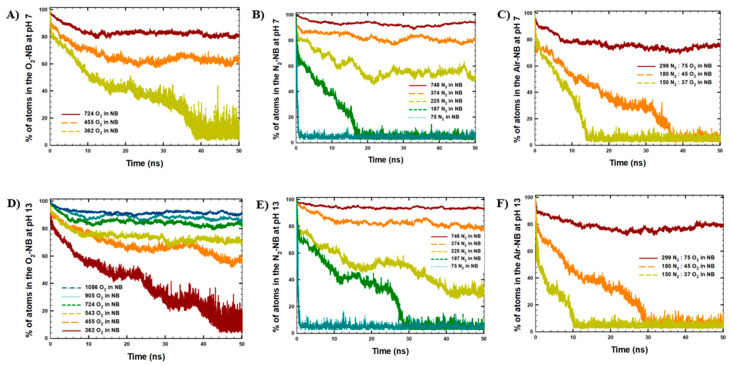
Cluster analysis with the maximum number of atoms in the NBs: (**A**) O_2_-NB at pH 7, (**B**) N_2_-NB at pH 7, (**C**) Air-NB at pH 7, (**D**) O_2_-NB at pH 13, (**E**) N_2_-NB at pH 13, (**F**) Air-NB at pH 13.

The effect of gas type, gas density, and pH variations on the stability of NBs was analyzed based on the number of atoms remaining in the NBs (Figure 2) and H-bonding between NBs and H_2_O, averaged over the last 10 ns of the MD simulations (Table 3). In particular, the time evolution of the average number of atoms in NBs indicated the dissolution of atoms from the NBs to the simulation box within the simulated time (Figure 2). Similarly, the formation of H-bonds between NBs and H_2_O molecules indicated the solvation of NB gas molecules in water (Table 3).

The reproducibility of the results of our MD simulations was validated by performing MD runs three times for the systems with 724 O_2_ molecules and 748 N_2_ molecules at pH 7 and pH 13 (Table S1, Figure S2). The results of the three runs were in agreement, indicating good reproducibility of our MD simulations. In particular, the results showed that N_2_-NB was more stable than O_2_-NB, with the relative percentage of atoms remaining in the NBs in the last 10 ns of the simulations at values around 94% and 80%, respectively. Overall, this observation was in agreement with the gas solubility tendency, which shows a higher solubility of O_2_ gas in water at 25 °C (0.039 g/L), in comparison to the solubility of N_2_ gas in water (0.019 g/L).

Moreover, according to the results of cluster and H-bonding analyses shown in Figure 2 and Table 3, the NB gas type affected NB stability. In particular, a lower number of atoms remained in the NBs after 50 ns of the simulations with O_2_ molecules, in comparison to the NBs with N_2_ and air content. Similarly, a higher number of H-bonds was observed between NBs and water in the systems with O_2_ content, consistent with the lower stability of O_2_-NB, as noted previously.

Moreover, NB stability decreased with decreasing concentrations of gas in the initial NBs for all types of gas (N_2_, O_2_, air model) under study. This observation was indicated by a lower number of atoms remaining in the NBs after 50 ns of the simulations at lower gas concentrations, with higher gas dissolution from the NBs (Table S2). For example, at pH 13, the decrease in O_2_ gas concentration in the NBs from 1086 molecules to 362 molecules resulted in a decrease in the average number of atoms that remained in the NBs at the end of the simulations from 1981 ± 15 atoms (91 ± 1%) to 94 ± 38 atoms (13 ± 5%).

Similarly, for N_2_-NBs, the decrease in N_2_ gas concentration in NB from 748 molecules to 75 molecules resulted in a decrease in the average percentage number of atoms observed in the NBs at the end of the simulations from 93–94% to 5%. In addition, for air-NBs, the decrease in gas concentration in the NBs from 374 molecules to 187 molecules resulted in a decrease in the average percentage number of atoms in the NB at the end of the simulations from 74–79% to 4–5%. The apparent lower stability of air-NBs compared with N_2_ or O_2_ systems arises mainly from their smaller initial gas content. At comparable initial loading (~370 atoms), air- and N_2_-NBs display similar retention and H-bonding behavior, whereas O_2_-NBs are less stable. At lower initial gas densities (225 atoms), N_2_-NBs retained a higher fraction of gas atoms than air, which can be attributed to the higher solubility of O_2_ in water, facilitating gas loss from air bubbles. Our simulations were in agreement with the results of the MD study of Hewage et al. [16], which observed higher stability of O_2_-NB at higher initial gas density in NBs.

**Figure 3:**
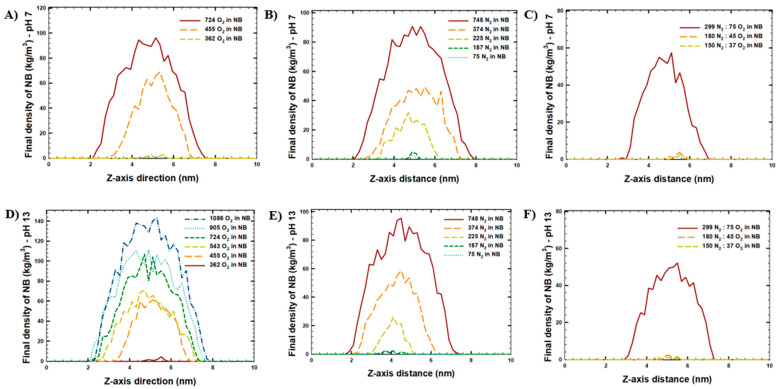
Density analysis of gas molecules in NBs observed at the end of the MD simulations (last frame): (**A**) O_2_-NB at pH 7, (**B**) N_2_-NB at pH 7, (**C**) Air-NB at pH 7, (**D**) O_2_-NB at pH 13, (**E**) N_2_-NB at pH 13, (**F**) Air-NB at pH 13.

Furthermore, considering the effect of alkalinity, no significant difference in the NB stability was observed for the NBs at pH 7 and pH 13. Small variations in the retained gas fraction may result from minor differences in interfacial water structure or gas distribution at different pH values, but remain within simulation uncertainty. The relatively large decrease in the retained fraction for the N_2_-NB with 225 molecules likely reflects their proximity to the dissolution threshold. In small bubbles, the strong curvature and high internal pressure make the system more sensitive to subtle interfacial differences between pH 7 and pH 13, rather than indicating a true pH-dependent effect. The results of the MD simulations were further studied via NB gas density analyses at the beginning of the simulations (Figure S3: initial NB density) and at the end of the simulations (Table 3, Figure 3).

According to Figure S3, the initial diameter of the NBs was 5 nm in all of the simulated systems. The NB diameter was determined from the linear number-density profile of gas atoms calculated for the final equilibrated frame. Only the atoms belonging to the NBs were considered. The diameter was defined as the distance between the two outermost positions where the gas density dropped to zero on opposite sides of the bubble center. Consistent with our previous observations (Figure 2), comparatively larger NB sizes and higher NB gas densities were observed at the end of the simulations at higher initial NB gas concentrations (Table 3, Figure 3). For example, the decrease in O_2_-gas concentration in the NBs at pH 13 from 1086 molecules to 362 molecules resulted in a decrease in the final NB diameter observed at the end of the simulation from 6.32 nm (with the maximum NB density of 145 kg/m^3^) to 1.0 nm (with the maximum NB density of 4 kg/m^3^). Similarly, for N_2_-NB at pH 7, the decrease in gas concentration in NB from 748 molecules to 75 molecules decreased the final NB diameter observed at the end of the simulation from 5.61 nm (with the maximum NB density of 92 kg/m^3^) to 0.40 nm (with the maximum NB density of 4 kg/m^3^). In addition, it should be noted that although the initial NB size was 5 nm, the final NB size increased at the highest initial NB gas concentration (1086 O_2_ molecules in NB) up to 6.32 nm. This observation indicates that although 91% of atoms remained in the NB, the presence of oversaturated gas in the NB would result in an increased size of the NB, as compared to the initial NB size. In some cases, such as for 362 O_2_-NB systems (10 ± 4% of atoms remained in the NB within a 1.21 nm diameter at pH 7, and 13 ± 5% of atoms remained in the NB within 1.00 nm diameter at pH 13) and for 187 Air-NB (5 ± 1% of atoms remained in the NB within 1.00 nm diameter at pH 7, and 4 ± 1% of atoms remained in the NB within 1.00 nm diameter at pH 13), smaller NBs may be denser and more compact, leading to a higher fraction of atoms despite a smaller diameter. Conversely, a larger apparent diameter may result from a more diffuse gas distribution near the interface.

**Figure 4:**
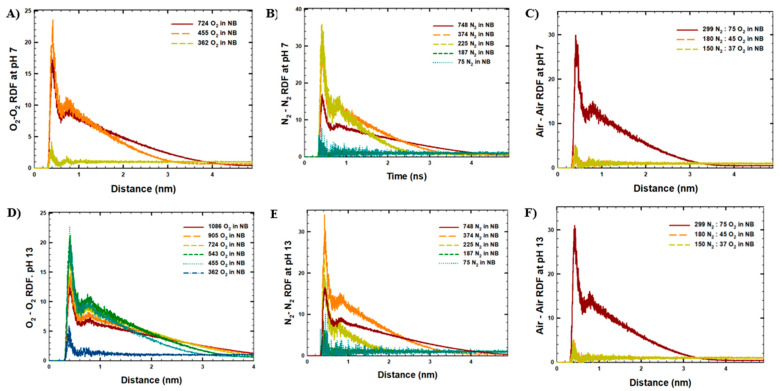
RDF analysis between gas molecules in the last 10 ns of MD runs in the simulated systems: (**A**) O_2_-NB at pH 7, (**B**) N_2_-NB at pH 7, (**C**) Air-NB at pH 7, (**D**) O_2_-NB at pH 13, (**E**) N_2_-NB at pH 13, (**F**) Air-NB at pH 13.

The results of the MD simulations were further studied in terms of RDF analyses on the interactions between gas molecules (Figure 4). As shown, the lowest RDF values (maximum peak around the value of 5 at 0.5 nm distance) were observed for the simulated systems with the lowest initial NB gas densities, either because the NB size was small or because of the lowest stability of NBs. Interestingly, for the highest initial NB gas densities, higher RDF interactions were observed at long-range distances (up to 4 nm distance), as the size of the final NBs was comparatively larger (5 nm and higher). In contrast, the highest maximum RDF peaks within 0.5 nm were observed for the NBs with a final size of 2–4 nm, such as the systems with 455 O_2_ or 225–374 N_2_ molecules in the NBs at pH 7 and pH 13.

Furthermore, the solvation of gas molecules was studied via RDF analysis of the interactions between gas and water molecules averaged among the last 10 ns of the MD simulations (Figure S4). According to Figures S4 and S5, as the initial NB gas density decreased, the NB–water and NB–OH^−^ ion interactions increased, indicating higher solvation of gas molecules in the systems with lower initial NB gas concentrations. This observation was consistent with the results of our cluster analysis, which showed low stability of NBs with low initial gas concentrations. Similarly, no effect of pH on NB stability was observed via RDF analysis in terms of gas solvation. The representative snapshots of the simulated systems visualized at the end of the simulations are shown in Figure S6.

### 3.2. Stability of Nanobubbles: Effect of Organic Compounds with Various Functional Groups

The effect of organic compounds with various functional groups and hydrophobicity (acetic acid, ethanol, n-hexane) was studied on O_2_-NBs and N_2_-NBs with high and low gas densities. Moreover, the synergistic effect of organic compounds and alkalinity was studied for O_2_-NBs with 362 molecules in the initial NBs. Finally, the impact of hexane molecules was examined on air-NBs with different gas densities in the initial NBs.

**Figure 5:**
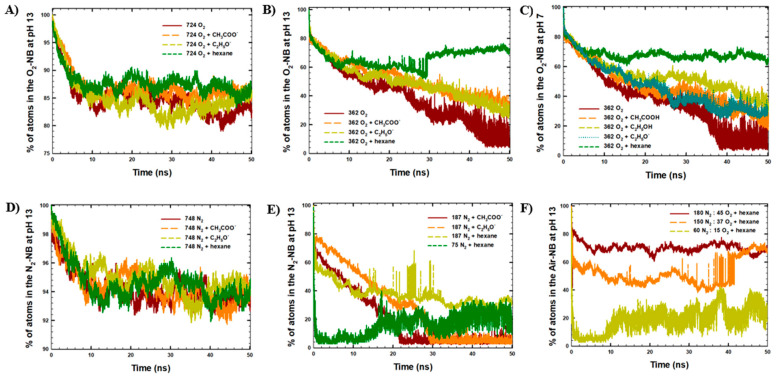
Cluster analysis with the maximum number of atoms in the NBs in the simulated systems with a NB and 100 organic molecules of acetic acid, ethanol, and hexane: (**A**) 724 O_2_ molecules in NB at pH 13, (**B**) 362 O_2_ molecules in NB at pH 13, (**C**) 362 O_2_ molecules in NB at pH 7, (**D**) 748 N_2_ molecules in NB at pH 13, (**E**) 187 and 75 N_2_ molecules in NB at pH 13, (**F**) 225, 187, and 75 molecules in Air-NB at pH 13.

The stability of NBs was first studied via cluster analysis and time-evolution of the number of atoms in the NB within the simulated time (Figure 5). The average number of atoms remaining in the NBs in the last 10 ns of the simulations was reported in Table 4. Similarly, solvation of gas molecules was studied via H-bond analysis between NB and H_2_O, averaged among the last 10 ns of the MD simulations (Table 3).

According to the results of cluster analysis (Figure 5A, Table 4), the presence of organic compounds slightly improved NB stability at comparatively high initial O_2_ gas concentration in the NBs (with 724 O_2_ molecules). In particular, the presence of organic molecules increased the average percentage of atoms remaining in the NBs in the last 10 ns of the simulations up to 84–86%, in comparison to 82% observed in the absence of organic molecules (Table 3).

In comparison, the presence of organic compounds significantly improved NB stability at a comparatively low initial O_2_ gas concentration in NBs (with 362 O_2_ molecules). In particular, at pH 13, the presence of CH_3_COO^−^, C_2_H_5_O^−^, and hexane increased the average percentage number of atoms remaining in the NBs from 13 ± 5% up to 37 ± 3%, 33 ± 3%, and 72 ± 1%, respectively (Figure 5B, Table 4). Similarly, the presence of CH_3_COOH_,_ C_2_H_5_OH, and hexane increased the average percentage of atoms remaining in the NB from 10 ± 4% up to 28 ± 3%, 32 ± 3%, and 67 ± 1%, respectively (Figure 5C, Table 4). Comparing the effects of three organic molecules, a significant decrease in H-bonding between O_2_ and water from 39 H-bonds (in the absence of organic molecules, Table 3) to 22–23 H-bonds was observed in the presence of hexane molecules (with a hydrophobic nature) at both pH conditions (Table 4). Interestingly, according to Figure 5B, a sharp increase in the percentage of atoms in NBs from 53% to 72% was observed at 28 ns of the MD run in the system with hexane molecules, suggesting adsorption of a group of O_2_ molecules back to the NBs. This observation indicated that among different organic molecules, hydrophobic molecules might increase the number of molecules in NBs, possibly serving as carriers of gas molecules due to strong hydrophobic interactions with gas molecules, which will be discussed further in detail with RDF analysis.

Furthermore, according to Figure 5D, the presence of organic compounds did not affect NB stability at comparatively high initial N_2_ gas concentrations in NBs (with 748 N_2_ molecules), with 93–94% of atoms remaining in the NBs in the absence and presence of organic molecules (Table 3 and Table 4). Interestingly, according to Figure 5E, at extremely low initial N_2_ gas concentrations in NBs (with 187 N_2_ and 75 N_2_ molecules), only hydrophobic hexane enhanced the percentage of atoms in NBs from 5% to 25–30%, with no significant effect on H-bonding between gas and water molecules.

Furthermore, according to Figure 5F, the presence of hexane molecules increased the percentage of atoms in Air-NB with 187 and 225 gas molecules from 4 ± 1% and 5 ± 1% to 65 ± 8% and 68 ± 3%, respectively. In addition, at an extremely low initial gas concentration in Air-NB (with 75 molecules), 27 ± 7% of atoms remained in the NB. Moreover, in the system with 187 gas molecules in Air-NB, the adsorption mechanism of air molecules back to NBs was observed at 42 ns of the MD run, indicated by a sharp increase in the percentage of atoms in NB from 44% to 64%.

The intermolecular interactions were analyzed via RDF analysis averaged among the last 10 ns of MD simulations (Figures S7–S9). According to Figure S7A,D, the presence of organic molecules did not significantly affect the interactions between gas molecules at high initial NB gas concentrations, with 724 O_2_ and 748 N_2_ molecules. In comparison, according to Figure S7B,C,E, at low initial NB gas concentrations, with 362 O_2_, 187, and 75 N_2_ molecules, the presence of organic molecules increased interactions between gas molecules, indicated by elevated peaks at 0.4 nm distance on RDF plots. It should be noted that among organic compounds, hexane molecules significantly increased interactions between gas molecules at low initial NB gas concentrations. Similarly, the presence of hexane increased interactions between gas molecules in the systems with Air-NB (Figure S7F). Furthermore, according to Figure S8, the presence of organic molecules affected the solvation of gas molecules in water. In particular, hexane molecules significantly decreased the interactions between gas and water molecules at low initial NB gas concentrations.

The RDF interactions between gas molecules and organic compounds were further studied, as shown in Figure S9. The results showed enhanced interactions between gas and hexane molecules, indicated by elevated RDF peaks at all gas concentrations and different pH conditions. This observation is in agreement with our previous conclusion from the cluster analysis (Figure 5) that hexane molecules absorbed gas molecules back to the NBs due to high hydrophobicity and high affinity for binding to NB gas. Moreover, the interactions between gas molecules and two other organic compounds (acetic acid and ethanol) were affected by the pH conditions, as shown in Figure S9B,C. Particularly, comparatively lower interactions between organic compounds and gas molecules were observed under alkaline conditions, correlated with the presence of a negative charge on the deprotonated forms of acetic acid and ethanol molecules at pH 13.

Overall, the results of the cluster and RDF analyses showed that hydrophobic organic molecules would enhance the stability of neutrally charged hydrophobic NBs by adsorption of gas molecules from water to NBs. Conversely, hydrophilic organic compounds would not significantly contribute to NB stability due to poor interactions with gas molecules, especially under alkaline conditions with charged organic molecules. Our results were in line with the results of previous MD studies, which showed high interactions and adsorption of neutral NBs, like N_2_-NB, with hydrophobic domains [19,20], and low interactions of NBs with hydrophilic surfaces [21].

**Figure 6:**
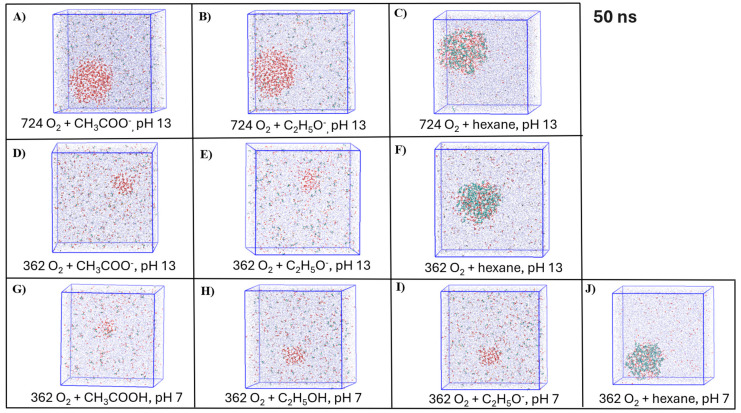

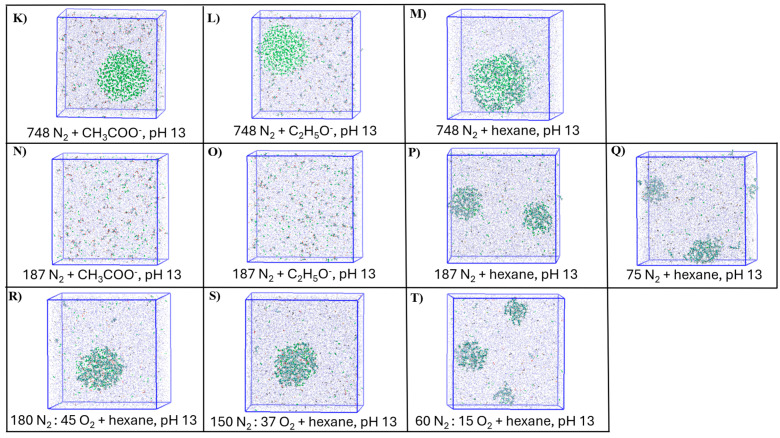
Representative snapshots of the simulated systems depicted at the end of the simulations: (**A**–**J**) O_2_-NB and organic molecules, (**K**–**Q**) N_2_-NB and organic molecules, (**R**–**T**) Air-NB and hexane molecules. VMD Coloring methods: (1) CPK opaque: red—Oxygen, green—Nitrogen, black—OH^−^, orange—Na^+^; (2) CPK transparent: blue—water molecules; (3) Licorice: organic compounds: cyan—carbon, white—hydrogen, red—oxygen.

The representative snapshots of the visualized simulated systems with NBs and organic molecules depicted from the last frame of the MD simulations are shown in Figure 6. Based on the visualization of the simulated systems with hexane molecules, the formation of several clusters of hexane and gas molecules was observed at low initial NB gas densities (Figure 6P,Q,T). This observation supports our results of the cluster analysis (Figure 5E,F, Table 4) and shows the potential stabilization effect of the organic compound–gas aggregation on the stability of NBs with a neutral charge.

## 4. Conclusions

In this study, the stability of nanobubbles (NBs) was studied under various conditions using MD simulations. Our research focused on understanding the factors affecting NB stability, including gas type, gas density, alkalinity, and the presence of organic compounds with different functional groups and hydrophobicity.

The results of this study showed that the stability of NBs varied significantly depending on the type of gas present in the bubbles. N_2_-NBs exhibited higher stability compared to oxygen O_2_-NBs, which can be attributed to the lower solubility of N_2_ gas in water. Moreover, decreasing the initial gas density in NBs led to decreased stability, indicating the importance of gas concentration for NB longevity. Interestingly, alkalinity did not show a significant effect on NB stability. Furthermore, the presence of organic compounds, such as acetic acid, ethanol, and hexane, influenced NB stability differently based on their hydrophobicity. Hydrophobic hexane molecules showed a significant stabilizing effect on NBs, especially at low initial NB gas concentrations, possibly by acting as carriers for gas molecules due to strong hydrophobic interactions.

Our results underscore the complex interplay between gas composition, density, and the presence of organic compounds in determining NB stability. These findings provide valuable insights into optimizing conditions for the effective use of NBs in various applications, ranging from environmental remediation to industrial processes. Future research could explore additional factors influencing NB stability, such as the effects of ionic strength and interfacial charge distribution, as well as the influence of shear flow. Experimental validation of these effects would further enhance our understanding of nanobubble dynamics under realistic conditions.

## Figures and Tables

**Table 1:** Simulated systems and the initial number of gas molecules in 5 nm NBs, solvated in 10 × 10 × 10 nm^3^ box with water (* 4:1 ratio of N_2_:O_2_ was used for the air-NB model). The average number of water molecules in the simulation boxes was 30,000 molecules.

pH	NB Gas	Initial Number of Molecules in NB					
pH = 7	O_2_-NB	724	455	362			
	N_2_-NB	748	374	225	187	75	
	* Air-NB		374	225	187		
pH = 13	O_2_-NB	1086	905	724	543	455	362
	N_2_-NB		748	374	225	187	75
	* Air-NB			374	225	187	

**Table 2:** Simulated systems with NBs and organic compounds and the initial number of molecules in the simulation boxes. The average number of water molecules in the simulation boxes was 30,000 molecules.

NB—Type	Simulated System	CH_3_COO^−^/CH_3_COOH	C_2_H_5_O^−^/C_2_H_5_OH	C_6_H_14_	Na^+^	OH^−^
724 molecules in O_2_-NB (pH 13)	724 O_2_ + CH_3_COO^−^	100	-	-	160	60
	724 O_2_ + C_2_H_5_O^−^	-	100	-	160	60
	724 O_2_ + hexane	-	-	100	60	60
362 molecules in O_2_-NB (pH 13)	362 O_2_ + CH_3_COO^−^	100	-	-	160	60
	362 O_2_ + C_2_H_5_O^−^	-	100	-	160	60
	362 O_2_ + hexane	-	-	100	60	60
362 molecules in O_2_-NB (pH 7)	362 O_2_ + CH_3_COOH	100	-	-	0	0
	362 O_2_ + C_2_H_5_OH	-	100	-	0	0
	362 O_2_ + C_2_H_5_O^−^	-	100	-	100	0
	362 O_2_ + hexane	-	-	100	0	0
748 molecules in N_2_-NB (pH 13)	748 N_2_ + CH_3_COO^−^	100	-	-	160	60
	748 N_2_ + C_2_H_5_O^−^	-	100	-	160	60
	748 N_2_ + hexane	-	-	100	60	60
187 molecules in N_2_-NB (pH 13)	187 N_2_ + CH_3_COO^−^	100	-	-	160	60
	187 N_2_ + C_2_H_5_O^−^	-	100		160	60
	187 N_2_ + hexane	-	-	100	60	60
75 molecules in N_2_-NB (pH 13)	75 N_2_ + hexane	-	-	100	60	60
180 N_2_ + 45 O_2_ molecules in NB (pH 13)	180 N_2_:45 O_2_ + hexane	-	-	100	60	60
150 N_2_ + 37 O_2_ molecules in NB (pH 13)	150 N_2_:37 O_2_ + hexane	-	-	100	60	60
60 N_2_ + 15 O_2_ molecules in NB (pH 13)	60 N_2_:15 O_2_ + hexane	-	-	100	60	60

**Table 3:** Percentage of atoms observed in the NBs and H-bonds observed between NBs and H_2_O, at the end of the simulations, * averaged among the last 10 ns; and NB diameter observed at the end of the MD runs.

NB-Gas Type	Number of Molecules in the Initial NB	* % of Atoms in the NB: Last 10 ns		* H-bonds Number, NB-H_2_O: Last 10 ns		NB Diameter (nm): Last Frame	
		pH 7	pH 13	pH 7	pH 13	pH 7	pH 13
O_2_	1086 O_2_	-	91 ± 1%	-	37 ± 6	-	6.32 nm
	905 O_2_	-	87 ± 1%	-	36 ± 6	-	6.32 nm
	724 O_2_	82 ± 1%	82 ± 1%	34 ± 6	34 ± 6	4.93 nm	5.21 nm
	543 O_2_	-	71 ± 1%	-	32 ± 6	-	4.71 nm
	455 O_2_	62 ± 2%	58 ± 3%	31 ± 6	33 ± 6	3.82 nm	3.61 nm
	362 O_2_	10 ± 4%	13 ± 5%	39 ± 6	39 ± 6	1.21 nm	1.00 nm
N_2_	748 N_2_	93 ± 1%	94 ± 1%	19 ± 5	18 ± 5	5.61 nm	5.39 nm
	374 N_2_	79 ± 1%	80 ± 1%	15 ± 4	15 ± 4	4.40 nm	3.90 nm
	225 N_2_	55 ± 3%	30 ± 2%	13 ± 4	16 ± 4	2.79 nm	2.20 nm
	187 N_2_	4 ± 1%	5 ± 2%	15 ± 4	15 ± 4	0.40 nm	1.40 nm
	75 N_2_	5 ± 1%	5 ± 1%	6 ± 3	6 ± 3	0.40 nm	0.40 nm
Air model	299 N_2_:75 O_2_	74 ± 1%	79 ± 1%	17 ± 4	16 ± 4	4.20 nm	3.89 nm
	180 N_2_:45 O_2_	5 ± 2%	5 ± 1%	20 ± 5	20 ± 5	0.60 nm	0.80 nm
	150 N_2_:37 O_2_	5 ± 1%	4 ± 1%	16 ± 4	16 ± 4	1.00 nm	1.00 nm

**Table 4:** Percentage of atoms observed in the NBs and H-bonds observed between NBs and H_2_O, at the end of the simulations, averaged among the last 10 ns.

NB-Gas Type & pH	Simulated System	% of Atoms in the NB: Last 10 ns	H-Bonds Number, NB-H_2_O: Last 10 ns
724 O_2_ in NB at pH 13	724 O_2_ + CH_3_COO^−^	86 ± 1%	31 ± 6
	724 O_2_ + C_2_H_5_O^−^	84 ± 1%	33 ± 6
	724 O_2_ + hexane	86 ± 1%	30 ± 6
362 O_2_ in NB at pH 13	362 O_2_ + CH_3_COO^−^	37 ± 3%	33 ± 6
	362 O_2_ + C_2_H_5_O^−^	33 ± 3%	34 ± 6
	362 O_2_ + hexane (pH 13)	72 ± 1%	22 ± 5
362 O_2_ in NB at pH 7	362 O_2_ + CH_3_COOH	28 ± 3%	34 ± 6
	362 O_2_ + C_2_H_5_OH	39 ± 4%	31 ± 6
	362 O_2_ + C_2_H_5_O^−^	30 ± 3%	35 ± 6
	362 O_2_ + hexane (pH 7)	67 ± 2%	23 ± 5
748 N_2_ in NB at pH 13	748 N_2_ + CH_3_COO^−^	93.3 ± 0.8%	19 ± 5
	748 N_2_ + C_2_H_5_O^−^	94.4 ± 0.4%	18 ± 4
	748 N_2_ + hexane	93.6 ± 0.4%	18 ± 4
187 N_2_ in NB at pH 13	187 N_2_ + CH_3_COO^−^	5 ± 1%	16 ± 4
	187 N_2_ + C_2_H_5_O^−^	5 ± 1%	16 ± 4
	187 N_2_ + hexane	30 ± 3%	11 ± 4
75 N_2_ in NB at pH 13	75 N_2_ + hexane	25 ± 5%	5 ± 2
180 N_2_ + 45 O_2_ in NB at pH 13	180 N_2_:45 O_2_ + hexane	68 ± 3%	12 ± 4
150 N_2_ + 37 O_2_ in NB at pH 13	150 N_2_:37 O_2_ + hexane	65 ± 8%	10 ± 3
60 N_2_ + 15 O_2_ in NB at pH 13	60 N_2_:15 O_2_ + hexane	27 ± 7%	6 ± 2

## Data Availability

All data supporting the findings of this computational study are reported in the manuscript and its Supplementary Material.

## Supplementary Materials

The following supporting information can be downloaded at: https://www.mdpi.com/article/10.3390/molecules30244712/s1, The Supplementary Material provides additional methodological and analytical details complementing the main text. It includes extended descriptions of the molecular dynamics simulation setups, force-field parameters, and system compositions for various nanobubble types under different pH and gas concentration conditions. Table S1 summarizes the average number of atoms retained in N_2_ and O_2_ nanobubbles during the final 10 ns of simulation runs, while Table S2 lists the initial and final atom counts, gas retention ratios, and hydrogen-bond statistics between nanobubbles and surrounding water molecules. Figures S1–S9 present representative snapshots of simulated systems, time-evolution plots of gas stability, density profiles, and radial distribution function (RDF) analyses characterizing gas–water, gas–ion, and gas–organic interactions. Together, these data provide detailed structural and dynamical insights supporting the discussions in the main manuscript.
