# Supplementary material for: Effect of Organic Compounds and Alkalinity on the Stability of Bulk Nanobubbles: A Molecular Dynamics Study
Source: Molecules. 2025 Dec 9;30(24):4712. doi: 10.3390/molecules30244712 (PMC12735740; doi:10.3390/molecules30244712)
Supplement: Supplementary file 1 [file molecules-30-04712-s001.zip › molecules-3970174-supplementary.pdf]

Supplementary Material for "Effect of Organic Compounds and Alkalinity on the Stability of Bulk Nanobubbles: A Molecular Dynamics Study"

Samal Kaumbekova[1], Serina Ng[2], Dhawal Shah[3], Ayaulym Amankeldiyeva[1], Sagyn Omirbekov[1,*], and Yanwei Wang[1,3,*]

[1]Center for Energy and Advanced Materials Science, National Laboratory Astana, Nazarbayev University, 53 Kabanbay Batyr Avenue, Astana, 010000, Kazakhstan

[2]Shijiazhuang Chang'An Yucai Building Materials Co., Ltd., Shijiazhuang 051430, China

[3]Department of Chemical and Materials Engineering, School of Engineering and Digital Sciences, Nazarbayev University, 53 Kabanbay Batyr Avenue, Astana, 010000, Kazakhstan.

Table S1. The number of atoms in NB observed at the end of the simulations, averaged among the last 10 ns of the MD simulations and averaged among three runs. The NB was identified as a contiguous cluster of gas atoms separated by less than 1.0 nm.

Simulated System	Run	Atoms in NB: last 10 ns	% in the NB: last 10 ns	Average %
748 N_2 molecules in initial NB at pH 7	#1	1395±7	93.3±0.4%	93.6±0.8%
	#2	1409±10	94.2±0.7%	
	#3	1398±13	93.5±0.8%	
748 N_2 molecules in initial NB at pH 13	#1	1411±13	94.3±0.8%	93.5±1.0%
	#2	1386±15	92.7±1.0%	
	#3	1398±6	93.5±0.4%	
724 O_2 molecules in initial NB at pH 7	#1	1191±15	82.2±1.1%	80.9±1.5%
	#2	1167±13	80.6±0.9%	
	#3	1157±19	79.9±1.3%	
724 O_2 molecules in initial NB at pH 13	#1	1190±17	82.2±1.2%	82.1±1.7%
	#2	1210±15	83.6±1.1%	
	#3	1166±18	77.9±1.2%	

Table S2. Number of atoms in the initial NB; the average number of atoms and the average percentage of atoms observed in the NB at the end of the simulations*; and H-bonds observed between NB-H_2O at the end of the simulations*, *averaged among last 10 ns.

pH	Number of molecules in NB	The initial number of atoms in NB	Atoms in NB: last 10 ns	% in the NB: last 10 ns	H-bonds number: NB-H_2O
7	724 O_2	1448	1191±15	82±1%	34±6
	455 O_2	910	563±14	62±2%	31±6
	362 O_2	724	69±27	10±4%	39±6
7	748 N_2	1496	1395±7	93±1%	19±5
	374 N_2	748	593±8	79±1%	15±4
	187 N_2	374	16±5	4±1%	15±4
	75 N_2	150	7±2	5±1%	6±3
7	299 N_2 : 75 O_2	748	557±10	74±1%	17±4
	180 N_2 : 45 O_2	450	23±7	5±2%	20±5
	150 N_2 : 37 O_2	374	17±5	5±1%	16±4
13	1086 O_2	2172	1981±15	91±1%	37±6
	905 O_2	1810	1574±14	87±1%	36±6
	724 O_2	1448	1190±17	82±1%	34±6
	543 O_2	1086	774±13	71±1%	32±6
	455 O_2	910	528±26	58±3%	33±6
	362 O_2	724	94±38	13±5%	39±7
13	748 N_2	1496	1411±13	94±1%	18±5
	374 N_2	748	598±10	80±1%	15±4
	225 N_2	450	137±9	30±2%	16±4
	187 N_2	374	18±6	5±2%	15±4
	75 N_2	150	7±2	5±1%	6±3
13	299 N_2 : 75 O_2	748	590±10	79±1%	16±4
	180 N_2 : 45 O_2	450	22±7	5±1%	20±5
	150 N_2 : 37 O_2	374	16±4	4±1 %	16±4

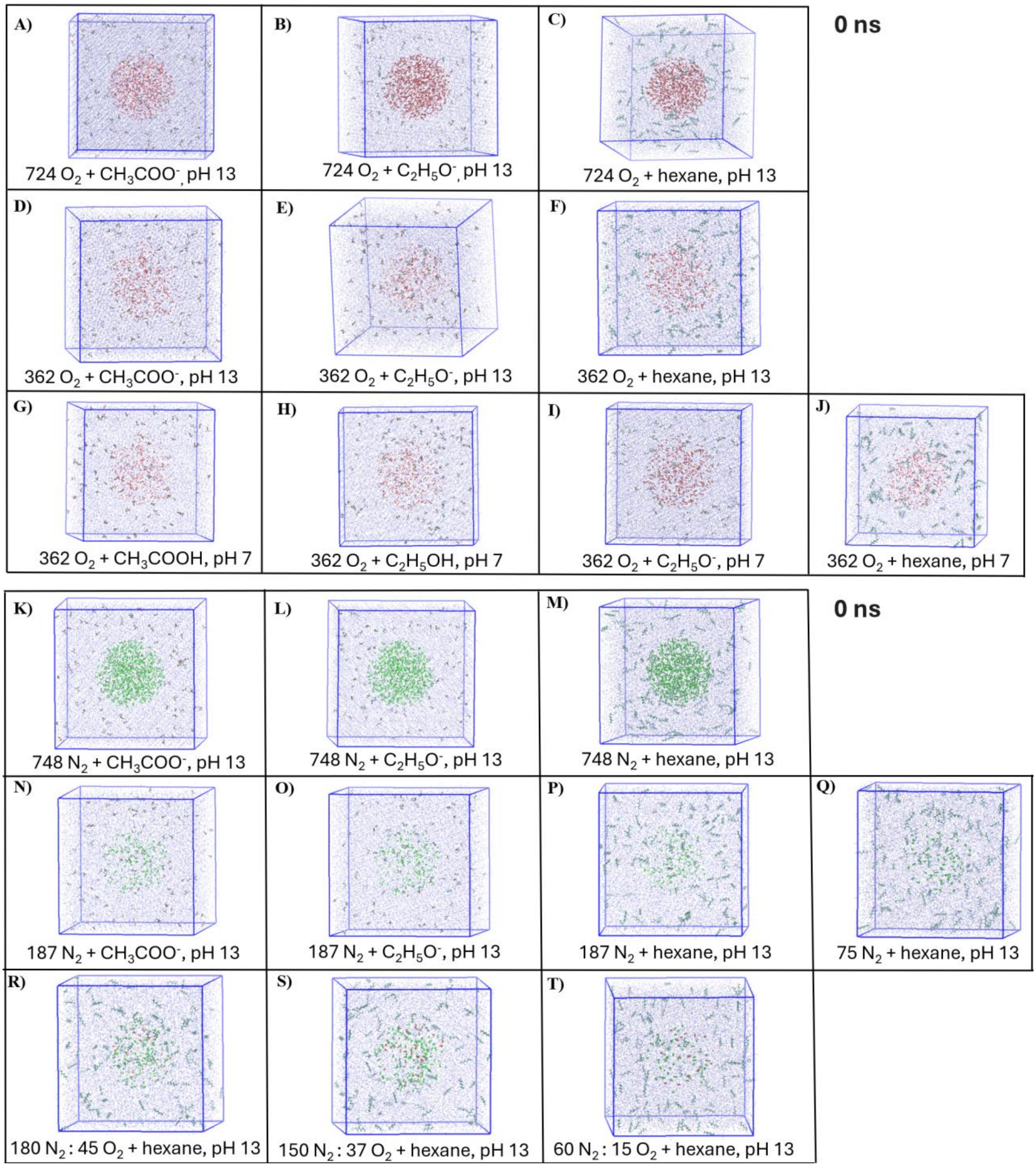

Figure S1. Representative snapshots of the simulated systems depicted at the beginning of the simulations: (A-J) O_2-NB and organic molecules, (K-Q) N_2-NB and organic molecules, (R-S) Air-NB and hexane molecules. *VMD Coloring methods: 1) CPK opaque: red–Oxygen, green–Nitrogen; 2) CPK transparent: blue–water molecules; 3) Licorice: organic compounds: cyan–Carbon, white–Hydrogen, red–Oxygen.*

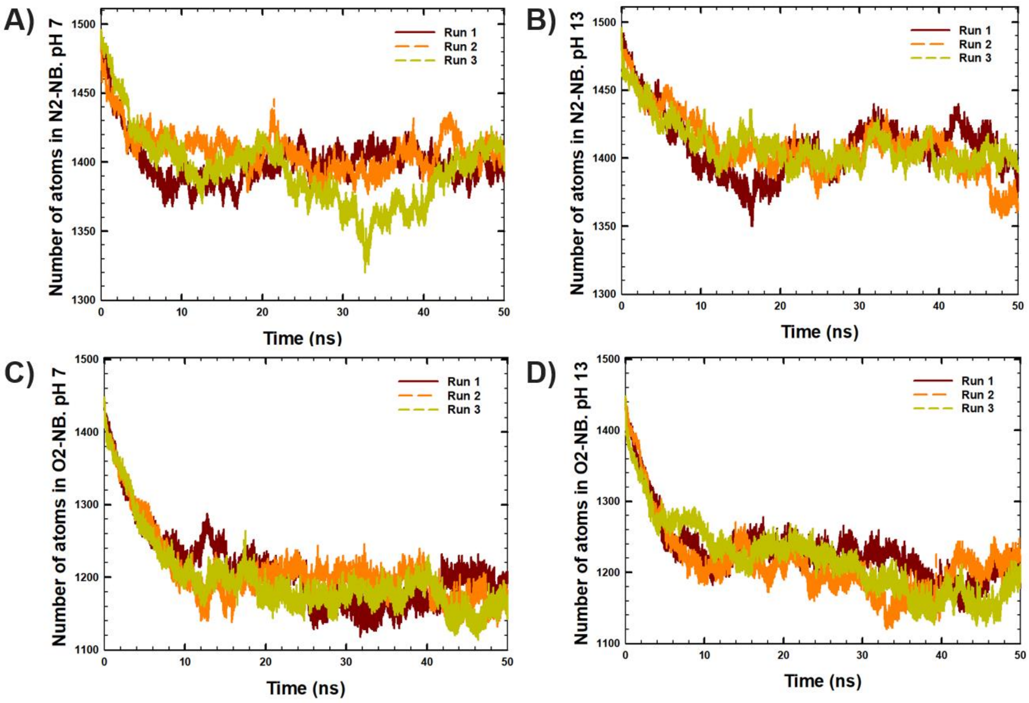

Figure S2. Time-evolution of the number of atoms in NB in three runs of the systems: (A) 748 N_2 molecules in NB at pH 7, (B) 748 N_2 molecules in NB at pH 13, (C) 724 O_2 molecules in NB at pH 7, (D) 724 O_2 molecules in NB at pH 13.

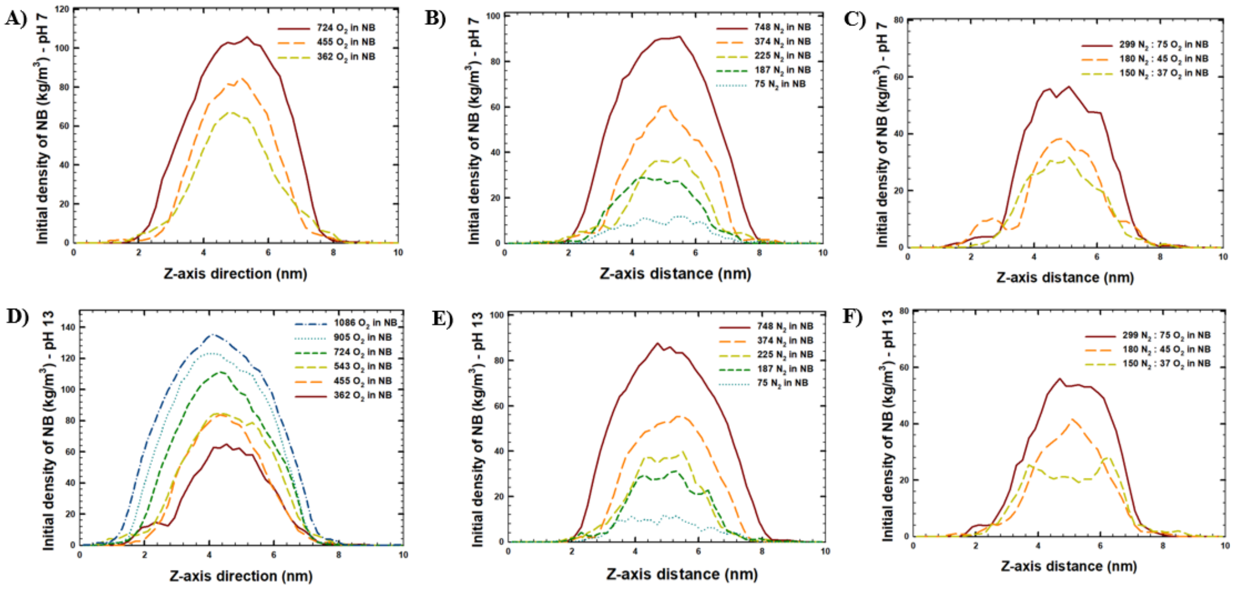

Figure S3. Density analysis of gas molecules in NB observed at the beginning of the MD simulations (first frame): (A) O_2-NB at pH 7, (B) N_2-NB at pH 7, (C) Air-NB at pH 7, (D) O_2-NB at pH 13, (E) N_2-NB at pH 13, and (F) Air-NB at pH 13.

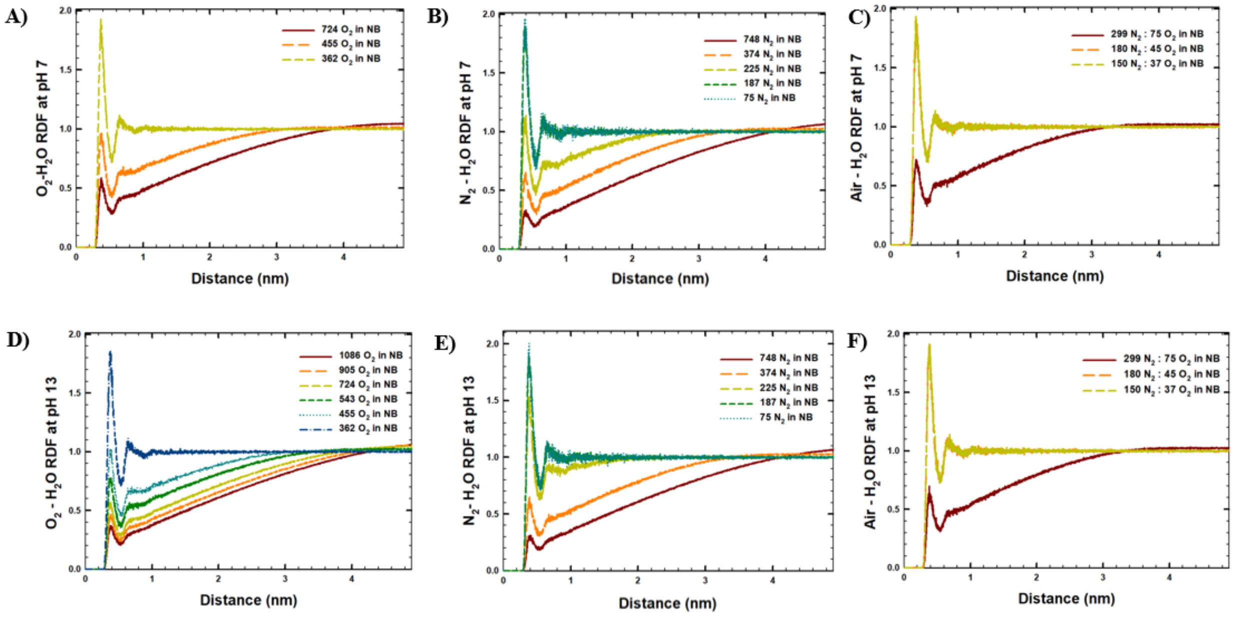

Figure S4. RDF analysis between gas molecules and water in the last 10 ns of MD run in the simulated systems: (A) O2-NB at pH 7, (B) N2-NB at pH 7, (C) Air-NB at pH 7, (D) O2-NB at pH 13, (E) N2-NB at pH 13, and (F) Air-NB at pH 13.

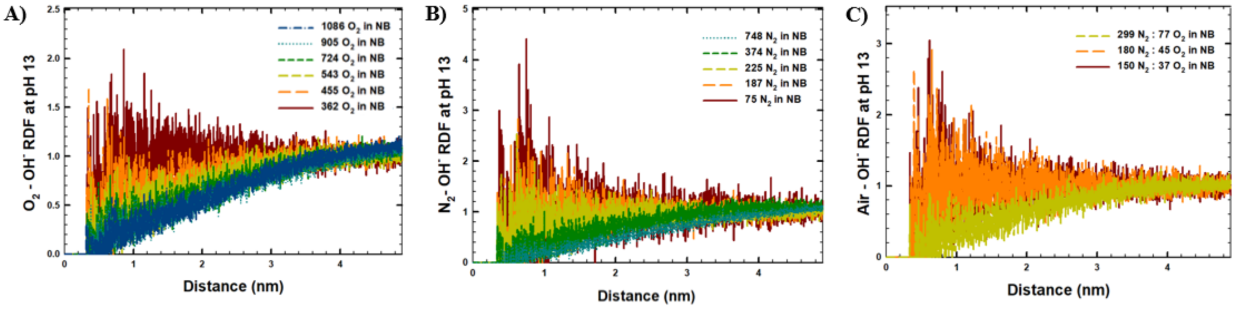

Figure S5. RDF analysis between gas molecules and OH^- ions in the last 10 ns of MD run in the simulated systems: (A) O2-NB at pH 13, (B) N2-NB at pH 13, and (C) Air-NB at pH 13.

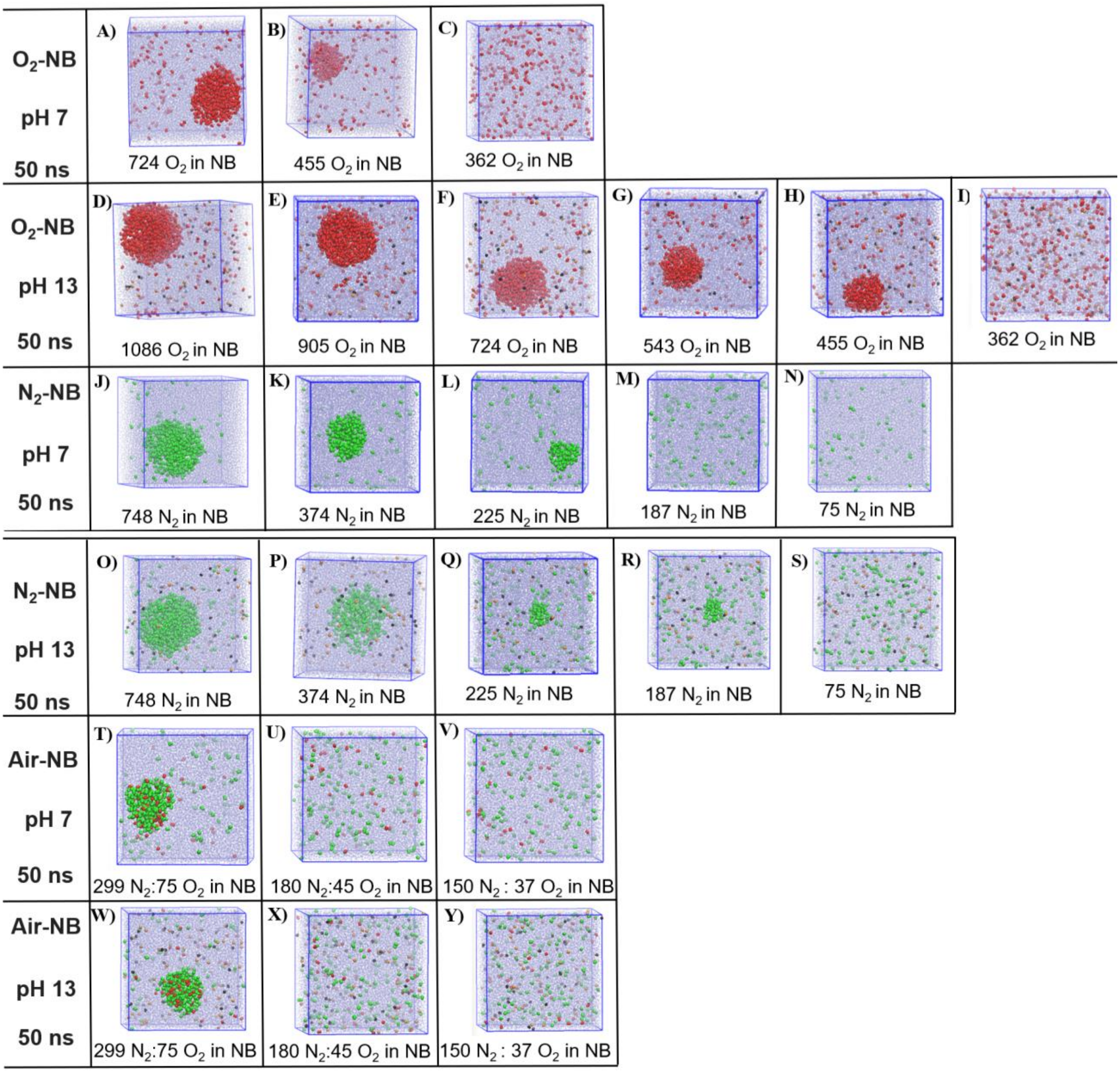

Figure S6. Representative snapshots of the simulation boxes depicted at the end of the MD run: (A-I) O_2-NB with different gas densities, (J-S) N_2-NB with different gas densities, (T-Y) Air-NB with different gas densities. *VMD Coloring methods: 1) Van der Waals representations: red - Oxygen, green – Nitrogen, orange = Na^+, black = OH^-; 2) Lines: blue – water molecules.*

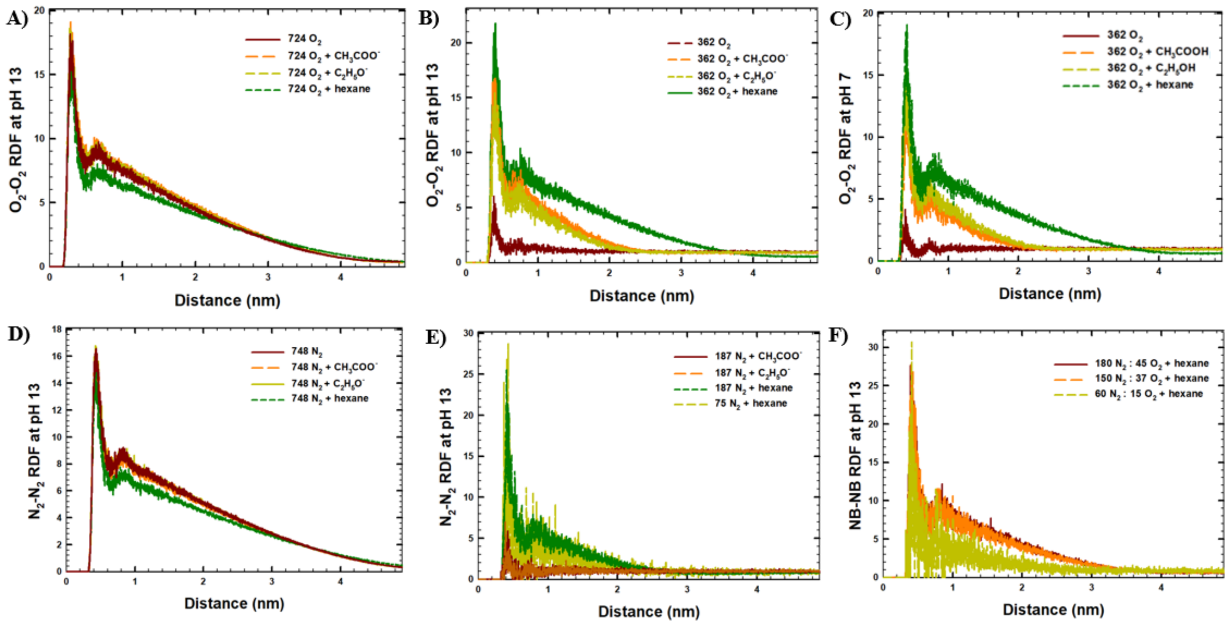

Figure S7. RDF analysis between gas molecules observed in the last 10 ns of MD run in the simulated systems: **(**A) 724 O_2 molecules in NB at pH 13, (B) 362 O_2 molecules in NB at pH 13, (C) 362 O_2 molecules in NB at pH 7, (D) 748 N_2 molecules in NB at pH 13, (E) 187 and 75 N_2 molecules in NB at pH 13, and (F) 225, 187, and 75 molecules in Air-NB at pH 13.

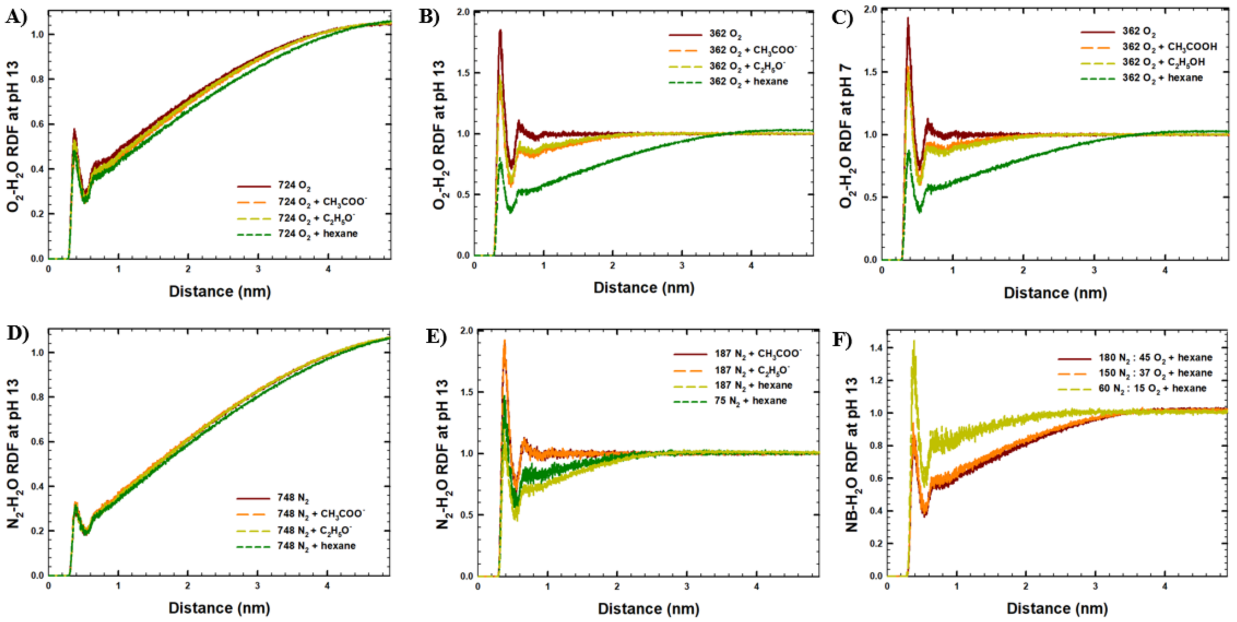

Figure S8. RDF analysis between gas molecules and water observed in the last 10 ns of MD run in the simulated systems: (A) 724 O_2 molecules in NB at pH 13, (B) 362 O_2 molecules in NB at pH 13, (C) 362 O_2 molecules in NB at pH 7, (D) 748 N_2 molecules in NB at pH 13, (E) 187 and 75 N_2 molecules in NB at pH 13, and (F) 225, 187, and 75 molecules in Air-NB at pH 13.

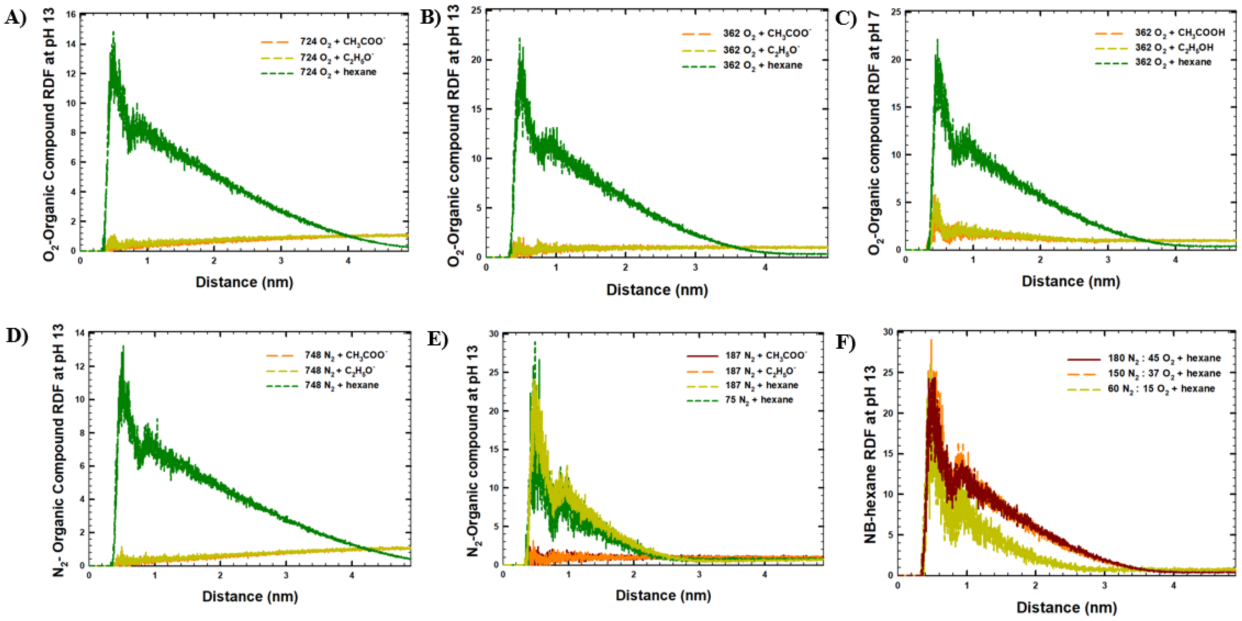

Figure S9. RDF analysis between gas molecules and organic compounds observed in the last 10 ns of MD run in the simulated systems: (A) 724 O_2 molecules in NB at pH 13, (B) 362 O_2 molecules in NB at pH 13, (C) 362 O_2 molecules in NB at pH 7, (D) 748 N_2 molecules in NB at pH 13, (E) 187 and 75 N_2 molecules in NB at pH 13, and (F) 225, 187, and 75 molecules in Air-NB at pH 13.
